# Arsenic and Ammonia

**Published:** 1890-10

**Authors:** 


### ARSENIC AND AMMONIA.

### REMARKABLE CONTRAST IN THE EFFECT OF TWO POISONS ON THE COMPLEXION.

The slow absorption of many poisons changes in some more or less modified form the complexion, but arsenic and ammonia show their effects about as quickly as any. The popular belief that arsenic clears the complexion has led many silly women to kill themselves with it in small continued doses.

It produces a waxy, ivory-like appearance of the skin during a certain stage of the poisoning, but its terrible after effects have become too well known to make it of common use as a cosmetic.

The effects of ammonia upon the complexion are directly the opposite to that of arsenic. The first symptom of ammonia poisoning, which appears among those who work in ammonia factories, is a discoloration of the skin of the nose and forehead. This gradually extends over the face until the complexion has a stained, blotched, and unsightly appearance. With people who take ammonia into their systems in smaller doses, as with their water or food, these striking symptoms do not appear so soon. The only effect of the poison that is visible for a time is a general unwholesomeness and sallowness of the complexion.

Many people are slowly absorbing ammonia poison without knowing it. The use of ammonia in the manufactures has greatly increased of late, and it is unquestionably used as an adulterant in certain food preparations. Official analyses have plainly showed its use even in such cheap articles of every day consumption as baking powders. The continued absorption of ammonia in even minute quantities as an adulterant in food, is injurious not merely from its effect upon the complexion, but because it destroys the coating of the stomach, and causes dyspepsia and kindred evils.

Professor Long, of Chicago, is authority for the statement that, if to fifty million parts of water there is one part of ammonia, the water is dangerous.

